# Cropping system-based fertilizer strategies for crop productivity and soil health under minimum tillage in grey terrace soil

**DOI:** 10.1016/j.heliyon.2024.e24106

**Published:** 2024-01-06

**Authors:** Md. Jahangir Alam, Mahammad Shariful Islam, A.T.M. Anwarul Islam Mondol, Habib Mohammad Naser, Nazmus Salahin, Md. Khairul Alam, Md. Mazadul Islam, Sanjida Akter, Zakaria Alam

**Affiliations:** a Soil Science Division, Bangladesh Agricultural Research Institute (BARI), Gazipur, Bangladesh; b Senior Scientific Officer, Regional Agricultural Research Station, BARI, Jashore, Bangladesh; c Tuber Crops Research Centre, BARI, Gazipur, Bangladesh; d Bangladesh Rice Research Institute (BRRI), Gazipur, Bangladesh; e Bangladesh Agricultural Research Council (BARC), Dhaka, Bangladesh

**Keywords:** Minimum tillage, Nutrient management, Crop productivity, Soil health, Four crops cropping system

## Abstract

A cropping system that is based on three or four crops is currently a widely favored option for augmenting crop productivity to address the escalating global food demand. However, the improper fertilizer management and undue tillage adversely impacts both the productivity of crops and the fertility of the soil. A research investigation was conducted on tillage and nutrient management within the mustard-mungbean-Transplanting aus (T.aus)-Transplanting aman (T.aman) cropping system to examine the impact of fertilizer packages and tillage techniques on the overall productivity of cropping systems, as well as the condition of the soil in grey terrace soil. The research included tillage techniques viz; minimum tillage (MT), conventional tillage (CT) and deep tillage (DT); while nutrient management; NM_1_: 100 % STB (Soil test based) following FRG (Fertilizer Recommendation Guide-2018), all from chemical fertilizer, NM_2_: 125 % of STB following FRG- 2018, all from chemical fertilizer, NM_3_: 100 % STB (80 % from chemical fertilizers and 20 % from cowdung), and NM_4_: Native fertility (no fertilization). A total of twelve treatments replicated three times following the factorial completely randomized design for three consecutive seasonal years (2018–19, 2019–20, and 2020–21). MT outperformed DT and CT in terms of crop yield, rice equivalent yield (REY), system productivity (SP), and production efficiency (PE). Moreover, NM_3_ exhibited enhanced performance in terms of agricultural productivity measures. Field capacity (FC), soil organic matter (OM), microbial biomass carbon (MBC), microbial biomass nitrogen (MBN), and soil nutrients (N, P, K, S, Zn and B) observed an enhancement as a result of the implementation of tillage MT and nutrition package NM_3_. The investigation indicates that implementing minimum tillage (MT) coupled with an integrated plant nutrition system package (NM3) can assist in the improvement of soil and the enhancement of crop productivity.

## 1 Introduction

The escalating global population has raised concerns about food security. In densely populated regions such as South Asia, the decline in soil fertility due to intensive crop cultivation has prompted discussions on the need for sustainable crop production. The cropping intensity in this region has recently been raised to meet the growing demand for food from an increasing population. Similarly, in Bangladesh, cropping intensity has also been raised to 214 % [1] in order to address the demand for food and ensure nutritional security, as the average population growth rate over the past five years has been 1.14 % [2] and the per capita arable land is 0.047 ha [3]. Additionally, the rate at which agricultural land is decreasing per year is 1 % [4] as a result of urbanization and industrialization. The incorporation of short-duration crop varieties, extensive tillage, the use of high yielding and hybrid varieties, and the excessive use of chemical fertilizer practices have made it possible to convert single cropping and double cropping systems into three crops, and even four crops cropping systems.

However, the excessive tillage, unbalanced fertilizer techniques and crop residue removal lead to the degradation of soil physical health [5], the dispersion of soil nutrients, and also contribute in soil organic matter declining [6]. Presently, in Bangladesh, 30.1 % of the arable land has organic matter levels below 1.5 % [7]. Moreover, heavy soil cultivation practices such as conventional or deep tillage have the potential to impact the physical health of the soil. These practices can lead to an increase in soil bulk density and soil strength, while simultaneously reducing the water holding capacity, and fertility status of the soil, specifically in terms of soil organic carbon and microbial biomass carbon [8,9]. Conversely, minimal or no tillage techniques have been found to mitigate the increasing soil bulk density [10] and penetration resistance [5]. Additionally, these practices can contribute to an increase in soil moisture content and water retention capacity [11]. Furthermore, minimal soil disturbance has been shown to enhance soil organic carbon [12] and microbial biomass carbon and nitrogen through microbial activity [13].

The recommended approach for meeting the nutritional requirements of crops, enhancing soil health, and promoting overall crop yield in a sustainable manner is the combined utilization of organic and mineral fertilizers [14]. For instance, the combinations contribute to the improvement of various physical properties of the soil, such as the reduction of bulk density [15], the decrease in penetration resistance [16], the enhancement of moisture retention [17], and the elevation of fertility levels, as evidenced by the rise in soil organic carbon [18]. Incorporating a higher number of crops into the cropping system leads to an enhancement in system productivity [19] and facilitates the uptake of residual nutrients [20].

Thus, tillage practices such as minimum tillage, along with the application of Integrated Plant Nutrition System (IPNS)-based fertilizer management, emerge as a compelling option for the enhancement of soil health. Various methods of tillage and residue management have been employed in order to enhance soil properties and improve crop yield in triple crop cropping systems [9,[21], [22], [23]]. The integration of tillage with organic and inorganic fertilizer strategies has also been investigated and indicated that the tillage, crop residue, and organic amendments have a positive impact on crop productivity and soil fertility [24]. However, there is a lack of research on the effects of these management practices in the context of four crop based cropping systems. While there have been studies on chemical fertilizer management for four crop-based systems [25,26], as well as comparisons study of system productivity between four crop and three crop cropping systems [20,27]. Therefore, the present study aims to examine the influence of tillage and organic-based nutrient management on cropping system productivity, as well as the effects of these treatments on soil health improvement.

## 2 Materials and methods

### 2.1 Statement of study site

During the three sequential growing periods of 2018–19, 2019–20, and 2020–21, the investigation regarding the cultivation regimen encompassing mustard–mungbean–T. aus–T. aman was carried out at agro-ecological zone (AEZ) 28, situated in the central region of the Madhupur tract. The experimental site was positioned at an approximate latitude of 23° 59′ 14″ N and a longitude of 90° 24′ 18″ E, with an average elevation of 8.4 m above the mean sea level. According to Ref. [28], the soils under the USDA Soil Taxonomy's Inceptisols order, Aquepts suborder, Grey terrace soils family and Chhiata series. According to Ref. [29] the soil is classified as Gleyic Luvisols Cutanine and Gleyic Alisols Cutanine.

### 2.2 Weather conditions of study area during crop cultivation period

**Fig. 1:**
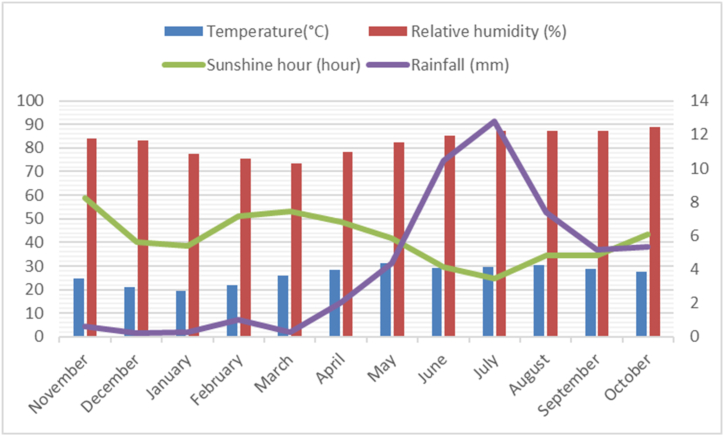
Monthly average temperature, relative humidity, sunshine hour and rainfall in experimental area at crop growing period.

The monthly temperature, relative humidity, sunshine hour and rainfall data of three-year growing period were collected from nearest weather station under the ministry of defense (Bangladesh Meteorological Department) and estimate mean value three years (Fig. 1). During crop growing period the mean temperature (°C) was remained lower from late November to mid-March with virtually dry condition (little rainfall in February). The maximum mean temperature and relative humidity were in the month from May to August and July–October, respectively. The maximum mean rainfall (mm) had been occurred during late May to Mid-July whereas the maximum sunshine hour was from February–April and mid-October to late November.

### 2.3 Experimental design and treatments

In this investigation, four distinct packages of nutrient management were employed in conjunction with three tillage operations. The tillage operations were namely, minimum tillage (MT) (4–6 cm depth), conventional tillage (CT) (8–10 cm depth), and deep tillage (DT) (15–20 cm depth). The nutrient management were viz. NM_1_ = 100 % STB (soil test based) dose following FRG [30], all from chemical fertilizer, NM_2_ = 125 % of STB (soil test based) dose following FRG- [30], all from chemical fertilizer, NM_3_ = 100 % STB dose (80 % from chemical fertilizers and 20 % from cowdung), and NM_4_ = Native fertility (no fertilization). A Randomized Complete Block (RCB) design, incorporating a two-factor approach, was utilized in order to generate twelve treatment combinations. These combinations were further divided into three replications, resulting in a total of thirty-six plots. Each individual plot was measured to be 5 m by 4 m in size. A detail about tillage operations and fertilizer dose as well as fertilization timings are described in Table 1.

**Table 1:** Description of cropping system with tillage, fertilizer dose and timings of fertilizer application.

Treatments		Cropping system			
		Mustard	Mungbean	T. aus	T. aman
Tillage practices	MT	With a power tiller operated seeder, minimum tillage was completed in a single pass.	One pass with a power tiller-operated seeder completed the minimum tillage	minimum tillage was done with spade	Minimum tillage was done with spade
	CT	4-5 passes using a power tiller machine	3-4 pass with power tiller machine	2-3 pass with power tiller machine	2-3 pass with power tiller machine
	DT	1-2 pass with chisel and single spading	1-2 pass with chisel and single spading	1-2 pass with chisel and single spading	1-2 pass with chisel and single spading
Nutrient management	NM_1_	Fertilizer dose: N_101_, P_15_, K_54_ S_12_ Zn_0.4_ and B_1.0_ kg ha^−1^	Fertilizer dose: N_20_, P_15_, K_32_ S_14_ Zn_0.4_ and B_1.0_ kg ha^−1^	Fertilizer dose: N_84_, P_4_, K_36_ S_8_ and Zn_0.2_ kg ha^−1^	Fertilizer dose: N_101_, P_4_, K_48_ S_11_ and Zn_0.13_ kg ha^−1^
	NM_2_	Fertilizer dose: N_126_, P_19_, K_67_ S_15_ Zn_0.5_ and B_1.25_ kg ha^−1^	Fertilizer dose: N_25_, P_19_, K_40_ S_17_ Zn_0.5_ and B_1.25_ kg ha^−1^	Fertilizer dose: N_105_, P_5_, K_45_ S_10_ and Zn_0.25_ kg ha^−1^	Fertilizer dose: N_126_, P_5_, K_60_ S_14_ and Zn_0.16_ kg ha^−1^
	NM_3_	Fertilizer dose: N_81_, P_12_, K_43_ S_10_ Zn_0.3_ and B_0.8_ kg ha^−1^ and cowdung 4.0 t ha^−1^	Fertilizer dose: N_16_, P_10_, K_25_ S_11_ and B_0.9_ kg ha^−1^ and cowdung 3.3 t ha^−1^	Fertilizer dose: N_67_, P_3_, K_29_ S_6_ and Zn_0.2_ kg ha^−1^ and cowdung 0.8 t ha^−1^	Fertilizer dose: N_81_, P_3_, K_38_ S_9_ and Zn_0.1_ kg ha^−1^ and cowdung 4.0 t ha^−1^
	NM_4_	No fertilizer	No fertilizer	No fertilizer	No fertilizer
Fertilization timing	NM_1_	Except for urea fertilizer, all fertilizers were applied during final land preparation. Two batches of urea application were made at 15 and 30 DAS.	At the end of the final land preparation, all fertilizers were applied. Two splits were treated with urea at 10 DAG and 25 DAG.	AT the last stage of land preparation, every fertilizer but urea fertilizer was used. Urea was administered in two installments at 10 DAT (at early tillering) and 25 DAT (before onset of panicles).	During final land preparation all fertilizers except urea fertilizer were applied. Urea was applied at 10 DAT (early tillering stage) and 20 DAT (tillering stage) and 35 DAT (before panicle initiation)
	NM_2_	Same as NM_1_	Same as NM_1_	Same as NM_1_	Same as NM_1_
	NM_3_	Cowdung was applied 3 days before sowing. Chemical fertilizers application method was same as NM_1_	Cowdung was applied 3 days before sowing. Chemical fertilizers application method was same as NM_1_	Cowdung was applied 5 days before sowing. Chemical fertilizers application method was same as NM_1_	Cowdung was applied 5 days before sowing. Chemical fertilizers application method was same as NM_1_

DAS = Days after sowing, DAT = Days after tran**s**planting, DAG = Days after germination, NM_1_ = 100 % STB (soil test based) dose, all from chemical fertilizer, NM_2_ = 125 % of STB (soil test based) dose, all from chemical fertilizer, NM_3_ = 100 % STB dose (80 % from chemical fertilizers and 20 % from cowdung), NM_4_ = Native fertility, T. aus = Transplanting aus and T.aman = Transplanting aman.

### 2.4 Sowing and transplanting of four crops cropping system and intercultural operations

The cropping system followed a sequence wherein Mustard (var. BARI Sharisha-14) was followed by Mungbean (var. BARI Mung-6), which in turn was followed by T. aus (var. BRRI dhan48), and finally T. aman (var. BRRI dhan62). The timing of sowing and transplanting remained consistent across all cropping seasons over a period of three years. For the cultivation of mustard, the process involved the sowing of seeds on the dates of November 8, 2018, November 7, 2019, and November 10, 2020. These seeds were sown with a spacing of 30 cm, using a continuous line technique. The harvesting of the mustard crop was done on February 9, 2019, February 8, 2020, and February 10, 2021, consecutively. In the case of mungbean cultivation, the seeds were sown on February 16, 2019, February 14, 2020, and February 16, 2021. Similarly, a spacing of 30 cm was maintained with the use of a continuous line technique. The crop was harvested on the 27th of April in the year 2019, the 26th of April in the year 2020, and the 28th of April in the year 2021. Regarding T. aus, seedlings were transplanted on the 2nd of May in the year 2019, the 3rd of May in the year 2020, and the 7th of May in the year 2021. The crop was harvested on the 22nd of July in the year 2019, the 22nd of July in the year 2020, and the 29th of July in the year 2021. For the transplantation of T. aman seedlings, the dates were July 28, 2019, July 29, 2020, and August 6, 2021. The harvesting of the crop took place on October 31, 2019, November 3, 2020, and November 14, 2021. The transplantation of both T. aus and T. aman seedlings was done maintaining a spacing of 20 cm by 20 cm. In the case of mustard, irrigations were applied during the application of fertilizer, with an additional irrigation during the pod formation stage. As for mungbean, irrigations were applied during the application of fertilizer. In the case of T. aus and T. aman, irrigation was applied only if necessary, as the growing period of the crop coincided with the rainfed season.

### 2.5 Soil sampling and analysis

After completion of the experiment, the soil samples (0–15 cm depth) were collected and dried at room temperature before being completely mixed, grinded, and sieved with a 2 mm sieve. Samples were then stored in plastic containers for further laboratory analysis. For instance, a modified Kjeldahl method [31] was used to quantify total N, while a colourimetric method [32] was used to determine available P. The soil organic matter was determined by wet oxidation method described by Ref. [33]. K and S were determined using the NH_4_OAC method [34] and the turbidimetric method [35], respectively. pH was determined using a glass electrode pH meter [36]. Using an atomic absorption spectrophotometer, micronutrients (Zn and B) were examined. Collected soil's particle size distribution was examined using the hydrometer method [37], and the USDA texture triangle was used to identify the textural class. The pressure plate method was used to measure soil moisture at different bars. By using a core sampler, the bulk density of the soil samples was ascertained [38]. Hand penetrometer Eijkelkamp (Netharlands) was used for penetration resistance. Chloroform fumigation-incubation was used to calculate the microbial biomass C [39]. A 40 g of soil, at 55 % of its water-holding capacity, were put into 50 ml glass beakers and fumigated for a day and evacuated, and then incubated for 10 days at 25 °C with 10 ml of 1 N potassium hydroxide (KOH). After titrating KOH with 1 N hydrochloric acid (HCl), carbon dioxide generation was measured [40]. According to Ref. [41], soil microbial biomass carbon was estimated by multiplying the mg of CO_2_ -C generated per kilogram of fumigated soil by an efficiency factor of 0.41. According to the procedure outlined by Ref. [42], microbial biomass nitrogen was calculated. Organic carbon (OC) and total organic carbon (TOC) were calculated with following formula.

OC=Organic matter/1.72. Where, 1.72 is the factor used to convert organic carbon from organic materials.

TOC (tha^−1^) = % OC* soil depth (cm)* bulk density (g cm^−3^).

Before launching the experiment, initial soil physico-chemical status of the study area was determined in the Laboratory of Soil Science Division, Bangladesh Agricultural Research Institute (BARI). Furthermore, the nutrient status of cowdung was also examined. Table 2a, Table 2b describes the initial physico-chemical characteristics of the soil and the nutritional status of cowdung.

**Table 2a:** Physico-chemical properties of initial soil during 2018.

Physical properties								
Soil depth (cm)	Particle size (%)			Textural class				
0–15	Sand	Clay	Silt	Clay loam				
	42.28	28.44	29.28					
	Moisture content (%)				Bulk density (g cm^−1^)	Penetration resistance (N cm^−2^)		
	0.3 bar (FC)	1.0 bar	2.0 bar	3.0 bar				
	30.20	24.89	21.34	19.12	1.51	267		
Chemical properties								
Soil depth (cm)	pH	OM	Total N	P (μg g^−1^)	K (meq 100 g^−1^)	S	Zn	B
		%				μg g^−1^		
0–15	5.54	1.14	0.058	12.3	0.12	11.9	1.19	0.15
Critical level	–	–	–	7.0	0.12	10	0.60	0.20
Interpretation	Acidic	Low	VL	Low	Low	Low	Low	Low

**Table 2b:** Nutrient content of decomposed cowdung.

Item	OM	N	P	K	S	Zn	B
	%						
Cowdung	9.8	0.81	0.87	0.53	0.35	0.15	0.01

### 2.6 Harvesting and data collections of yield and productivity

All crop**s** were harvested when they attained harvesting maturity. For each of the four crops, production of 1 m^2^ from each plot was recorded. Then the crop yield (t ha^−1^) was estimated by converting the 1 m^2^ area production into hectare of land. The following formula was used to calculate the Rice Equivalent Yield [43].

$$\text{REY}=\frac{\text{Mustard}\;\text{yield}\times \text{price}\;\text{of}\;\text{mustard}}{\text{price}\;\text{of}\;\text{rice}\;}+\frac{\text{mungbean}\;\text{yield}\times \text{price}\;\text{of}\;\text{mungbean}}{\text{price}\;\text{of}\;\text{rice}}+T.\;\text{aus}\;\text{yield}+T.\text{aman}\;\text{yield}$$

where, yield = t ha^−1^ and market price = Bangladesh Taka (BDT) kg.^−1^

The System productivity (SP) (t ha^−1^) was calculated with the formula: SP=Y_1_+Y_2_+Y_3_+Y_4_, where, Y_1_ denotes the first crop's yield, Y_2_ the second crop's yield, Y_3_ the third crop's yield, and Y_4_ the fourth crop's yield.

The $\text{Production}\;\text{efficiency}\;\left(\text{PE}\right)=\frac{Y1}{d1}+\frac{Y2}{d2}+\frac{Y3}{d3}+\frac{Y4}{d4}$, where crop yield was expressed as Y and cropping time as d. The PE is expressed with the unit of kg ha^−1^ day^−1^.

### 2.7 Statistical analysis

A year-by-year analysis of variance (ANOVA) and a pooled ANOVA spanning three years were conducted in order to assess the effects of tillage and fertilizer management on both productivity and soil health properties. The ANOVA was performed with the open-source R (R4.2.2 and RStudio). Tukey's Honestly Significant Difference (HSD) test at *p* < 0.05 were used to compare the mean values.

## 3 Results and discussion

### 3.1 Impact of tillage, nutrient management and their combination on crop yield

#### 3.1.1 Yearly effect of tillage on yield of cultivated crops

Tillage practices had no significant influence on the cultivated crops over the years (Table 3). In case of mustard, MT had the largest yield increasing trend (27.4 %), followed by CT (18.4 %) and DT (7.2 %). This might be result of accumulation of organic carbon through soil aggregate stabilization, and moisture conservation for less soil disturbance. On contrary, medium and heavy tillage degraded soil microbial abundance and soil water retention resulting depleted more nutrients. The results are in contrast to those of Salahin et al. [5], who found that strip tillage generated more mustard seed yield after three years experiment than conventional tillage under the mustard-boro-T. aman cropping system.

**Table 3:** Mean value of crop yield (t ha^−1^) in different tillage over three seasons (2018–2021).

Tillage operations	Mustard			Mungbean			T. aus			T. aman		
	2018–19	2019–20	2020–21	2018–19	2019–20	2020–21	2018–19	2019–20	2020–21	2018–19	2019–20	2020–21
MT	1.13	1.30	1.44	0.82	0.95	1.08	3.03	3.24	3.48	3.51	3.73	3.92
CT	1.14	1.27	1.35	0.91	1.02	1.06	3.21	3.38	3.47	3.72	3.79	3.90
DT	1.24	1.26	1.33	0.88	0.96	1.02	3.12	3.28	3.33	3.65	3.69	3.70
CV (%)	10.63	8.24	8.18	11.27	11.94	10.43	10.15	9.11	8.47	11.29	9.62	10.01
LS	NS	NS	NS	NS	NS	NS	NS	NS	NS	NS	NS	NS

MT = Minimum Tillage, CT=Conventional Tillage, DT = Deep Tillage, NS= Non significant, LS = Level of significant, CV=Co-efficient of variation, T. aus = Transplanting aus, T. aman = Transplanting aman.

Over a three-year period, there were no appreciable variations in the mungbean yield for various tillage techniques which is described in Table 3. In comparison to the first year, yield grew steadily. The largest yield increase was in MT method (31.7 %), followed by DT (16.5 %) and CT (15.9 %). This yield gap minimization may have occurred as lower water evaporation and nutrients mining from carbon riches soil owing to less disturbed soil. Oppositely, medium term and heavy tillage have disrupted surface soil pores resulting slow infiltration and ultimately nutrients leaching occurred through surface run off. The aforementioned results are consistent with those of Salahin et al. [9]'s study in mungbean under the wheat-mungbean-T. aman cropping system.

For three consecutive years, there was no discernible fluctuation in the seed yield of T. aus rice (Table 3). Table 3 demonstrated that yield was steadily improved and that the highest positive improvement eventuated in MT in percentage of 14.8 compared to the other two tillages (CT = 8.1 % and DT = 6.7 %) could occur because little soil disturbance increased organic matter in top soil due to soil aggregate stability as a result diversified microbes enhanced nutrient availability and root biomass and left-over nutrients using. Bhatt et al. [44]'s result informed that wheat had the highest seed production with no obvious difference between tillages and reduce tillage supports the claim.

For T. aman, there was no discernible difference in the amount of grain produced over the years (Table 3). Gradually, the yield was increased over the years and the maximum yield was increased in MT (11.7 %) followed by CT (4.8 %) and DT (1.4 %). The reason behind that, MT promotes more organic carbon to accumulate in the soil by dint of carbon decomposition and subsequent crop's long-term impact on soil carbon levels. Stored organic carbon helped to increase microbial biomass and nutrients concentration and utilize leftover nutrients. Rahman et al. [45]'s research also showed that minimal tillage performed noticeably better than conventional tillage in the boro-T. aman rice cropping system.

#### 3.1.2 Yearly effect of nutrient management on yield of cultivated crops

With the exception of NM_4_, mustard yield rose over time significantly (*p* ≤ 0.001) for all nutrient management practices (Table 4). In all years, NM_3_ significantly outperformed than other nutrient packages in terms of yield (first year = 1.48 t ha^−1^, second year = 1.66 t ha^−1^ and third year = 1.83 t ha^−1^), followed by NM_2_ (first year = 1.33 t ha^−1^, second year = 1.48 t ha^−1^ and third year = 1.62 t ha^−1^), NM_1_ (first year = 1.29 t ha^−1^, second year = 1.46 t ha^−1^ and third year = 1.60 t ha^−1^) and NM_4_ (first year = 0.59 t ha^−1^, second year = 0.51 t ha^−1^ and third year = 0.44 t ha^−1^). This phenomenon could potentially be attributed to the application of organic fertilizer, which augmented the organic carbon content within the uppermost layer of soil. Consequently, this facilitated the soil's capacity to retain a larger quantity of moisture, thereby fostering an increase in microbial population. This, in turn, resulted in a higher availability of nutrients for crops, achieved through the process of mineralization. Conversely, as no fertilizer was provided for the crops, a discernible downward trend in yield was observed for the NM_4_ treatment over the course of time. Additionally, due to the crops' extraction of nutrients from the soil's reserves, a deficiency in nutrients ensued. Reza et al. [46], who utilized both wholly inorganic fertilizer and a blend of organic and inorganic fertilizer, likewise achieved identical outcomes.

**Table 4:** Mean value of crop yield (t ha^−1^) in different nutrient managements over three seasons (2018–2021).

Nutrient management	Mustard			Mungbean			T. aus			T. aman		
	2018–19	2019–20	2020–21	2018–19	2019–20	2020–21	2018–19	2019–20	2020–21	2018–19	2019–20	2020–21
NM_1_	1.29 b	1.46 b	1.60 b	0.88 a	1.01 b	1.13 b	3.17 a	3.32 a	3.47 b	3.75 b	3.89 b	4.07 b
NM_2_	1.33 ab	1.48 b	1.62 b	0.92 a	1.06 b	1.17 b	3.46 a	3.64 a	3.82 ab	3.76 b	4.00 ab	4.15 ab
NM_3_	1.48 a	1.66 a	1.83 a	1.00 a	1.24 a	1.40 a	3.20 a	3.65 a	3.96 a	4.22 a	4.46 a	4.62 a
NM_4_	0.59 c	0.51 c	0.44 c	0.68 b	0.59 c	0.50 c	2.65 b	2.56 b	2.46 c	2.78 c	2.59 c	2.51 c
CV (%)	10.63	8.24	8.18	11.27	11.94	10.43	10.15	9.11	8.47	11.29	9.62	10.01
LS	***	***	***	***	***	***	***	***	***	***	***	***

NM_1_ = 100 % STB dose (chemical fertilizer), NM_2_ = 125 % of STB dose (chemical fertilizer), NM_3_ = IPNS (80 % chemical fertilizer+ 20 % organic fertilizer), NM_4_ = Native fertility, *** = 0.1 % level of significant, LS = Level of significant, CV=Co-efficient of variation, T. aus = Transplanting aus, T. aman = Transplanting aman.

With the exception of NM_4_, different nutrient management strategies significantly (*p* ≤ 0.001) influenced mungbean yield (Table 4). NM_3_ significantly performed the best yield (first year = 1.0 t ha^−1^, second year = 1.24 t ha^−1^ and third year = 1.40 t ha^−1^) than other nutrient management packages over the years followed by NM_2_ (first year = 0.92 t ha^−1^, second year = 1.06 t ha^−1^ and third year = 1.17 t ha^−1^), NM_1_ (first year = 0.88 t ha^−1^, second year = 1.01 t ha^−1^ and third year = 1.13 t ha^−1^) and NM_4_ (first year = 0.68 t ha^−1^, second year = 0.59 t ha^−1^ and third year = 0.5 t ha^−1^). The occurrence that took place could potentially be attributed to the fact that the crop effectively utilized the provided and remaining nutrients by means of a diverse microbial activity, while also making use of the stored soil moisture as an organic fertilizer to augment the accumulation of soil carbon and the capacity to retain soil water. As a consequence of the absence of supplementary nutrients and the extraction of stored nutrients by the crop, there was a significant decrease in the yield of the native fertility over a period of time. Based on the investigation conducted by Mollah et al. [47], it was observed that the IPNS approach (combination of chemical fertilizer and cowdung) yielded the highest amount of mungbean seeds compared to other alternative doses of chemical fertilizer.

T. aus rice grain yield was varied significantly (p ≤ 0.001) with different nutrient management over three years (Table 4). All nutrient management packages except NM_4_ yield was increased compare to first year. Among three nutrient management packages, there was no significant variation in first two years but in final years, NM_3_ package exhibited the maximum yield (3.96 t ha^−1^) which is statistically similar to NM_2_ (3.82 t ha^−1^) but significantly higher to NM_1_ (3.47 t ha^−1^) and NM_4_ (2.46 t ha^−1^) because soil carbon and nitrogen pool increased, due to organic fertilizer application resulting stabilized carbon degradation and less nutrients leached. Kaisar et al. [48] found same result in T. aus rice under integrated nutrient system.

During three successive seasons, nutrient management packages had a significant (*p* ≤ 0.001) impact on T. aman rice grain yield (Table 4). Over time, a rising trend in yield was seen for all nutrient management packages with the exception of NM_4_. In comparison to chemical fertilizer treatments (NM_1_ and NM_2_), and native fertility (NM_4_), organic based NM_3_ considerably produced the highest yield (first year: 4.22 t ha^−1^, second year: 4.46 t ha^−1^, and third year: 4.62 t ha^−1^). The rationales underlying the provision of organic fertilizer could be attributed to the stabilization of organic carbon and the enhancement of soil microbial biomass, consequently leading to an improvement in the availability of essential nutrients for crop growth. In the absence of any supplementary fertilizer to compensate for the NM_4_, crops experienced a deficiency in nutrients, resulting in a gradual decrease in yields (9.7 %). The findings presented by Salahin et al. [24] regarding the similarity of observations for T. aman rice align with the aforementioned outcomes.

#### 3.1.3 Pooled impact of years on crop yield

The production of mustard and mungbean, experienced considerable impacts, while the yield of T. aus and T. aman exhibited insignificant variations throughout the years (Table 5). A comparison between the initial and final year revealed yield increments of 17.1 %, 20.7 %, 9.9 %, and 5.8 % for mustard, mungbean, T. aus, and T. aman, respectively. The outcome may have arisen due to the availability of nutrients from both supplied sources and residuals from previous crops, which occurred through the process of mineralization facilitated by the diverse microbial population and the enhanced retention of soil moisture. Additionally, the reduced compaction of the soil, as a result of both tillage practices and the application of organic amendments, contributed to the accumulation of carbon in the surface soil. Furthermore, the annual fluctuations in weather conditions also played a role in this phenomenon. Das et al. [49] conducted a study over a span of four years, employing two different tillage methods and six distinct nutrient packages. The results of this investigation revealed a significant increase in yield, with a respective increment of 25.3 % and 56.95 % observed in maize and toria from the first year to the final year.

**Table 5:** Combined impact of year on yield (pooled) (t ha^−1^) of different crops.

Year	Mustard	Mungbean	T. aus	T. aman
2018–19	1.17 b	0.87 c	3.12 b	3.63
2019–20	1.28 ab	0.97 b	3.30 ab	3.74
2020–21	1.37 a	1.05 a	3.43 a	3.84
CV	15.8	6.51	14.8	12.4
LS	*	***	NS	NS

*** = 0.1 % level of significant, * = 5 % level of significant, NS= Non significant, LS = Level of significant, CV=Co-efficient of variation, T. aus = Transplanting aus, T. aman = Transplanting aman.

#### 3.1.4 Three-years impact of tillage on crop yield

When considering mustard, it can be observed that the amalgamation of three years' yield (as shown in Table 6) reveals that minimum tillage (MT) achieved the highest yield of 1.29 t ha^−1^. This was closely followed by deep tillage (DT) with a yield of 1.28 t ha^−1^, and conventional tillage (CT) with a yield of 1.25 t ha^−1^. MT has the potential to obtain the highest level of productivity during the second and third year in comparison to CT and DT, specifically in terms of the continuous accumulation of soil carbon, retention of soil moisture, and subsequent availability of nutrients through the mineralization process in minimally manipulated soil. In a separate study conducted by Chen et al. [50], it was also observed that reduced tillage resulted in a higher average yield of corn compared to conventional tillage when considering a four-year yield under an alfalfa-corn rotation. In the case of mungbean, the CT treatment exhibited a slightly higher yield of 1.0 t ha^−1^ compared to the MT treatment with a yield of 0.95 t ha^−1^, and also the DT treatment with a yield of 0.95 t ha^−1^, when the yield values from multiple years were combined (Table 6). These results align with the findings of DeJong-Hughes [51] in soybean under a soybean-corn system. The small additional yield obtained from the CT treatment, in comparison to the MT treatment, is insignificant considering that the MT treatment achieved the maximum yield in the final year through gradual yield improvement (Table 3). During the initial year in clay loam, the absence of adequate soil aeration and insufficient carbon accumulation resulted in a deficiency of essential nutrients for the crops. This issue could potentially be resolved by gradually increasing the soil's water retention capacity and depositing organic carbon over the course of several years, thereby compensating for the limited soil tillage. Additionally, excessive soil tillage may reduce the availability of nutrients through a process known as nutrients mining.

**Table 6:** Average yield (pooled) (t ha^−1^) of cultivated crops in different tillage over three seasons (2018–2021).

Tillage operations	Mustard	Mungbean	T. aus	T. aman
MT	1.29	0.95	3.25	3.72
CT	1.25	1.00	3.35	3.80
DT	1.28	0.95	3.34	3.68
CV (%)	8.94	11.24	9.24	10.30
LS	NS	NS	NS	NS

MT = Minimum Tillage, CT=Conventional Tillage, DT = Deep Tillage, NS= Non significant, LS = Level of significant, CV=Co-efficient of variation, T. aus = Transplanting aus, T. aman = Transplanting aman.

For T. aus, while MT ultimately attained the highest outcome (Table 3), CT outperformed the best than MT (3.35 t ha-^1^) and DT (3.34 t ha^−1^) in terms of yield across multiple growing seasons (Table 6). Das et al. [49] conducted research on the maize-toria cropping system and reported that the combined yield of maize over a four-year period was higher in the conventional tillage method compared to reduced tillage. This discrepancy was observed due to the superior performance of conventional tillage (CT) and deep tillage (DT) in the first year in terms of weed control, incorporation of residuals into the soil, and mitigation of soil compaction. However, continuous excessive tillage may have led to soil degradation and increased carbon decomposition, resulting in a decline in microbial populations and subsequently slowing down the availability of nutrients.

When combined, the results from the three years indicate that CT (3.80 t ha^−1^) achieved the best average yields compared to MT (3.72 t ha^−1)^ and DT (3.68 t ha^−1^) in T. aman rice (Table 6). In the first year, the yield of MT, when compared to CT and DT, may have been lower due to unfavorable soil and atmospheric conditions. Jiang et al. [52] conducted a study on the rice-ratoon rice cropping system and reported that the cumulative yield over four years is higher in the conventional cultivation method compared to the modern cultivation technique.

Among all four crops, the preferred method of tillage for mustard production has been minimum tillage, as indicated by the average yield over a period of three years. However, for mungbean, T. aus, and T. aman, conventional tillage has been determined to be the optimal choice. Although conventional tillage is considered the best option, there has been a gradual increase in crop yield with minimum tillage due to the successive improvement in soil health. If we had analyzed the production cost and net return of the crops, we would have been able to observe the true comparison between minimum tillage, conventional tillage, and deep tillage. Das et al. [49] found that conventional tillage resulted in a higher average yield than zero tillage, but zero tillage had a greater income under maize-toria cropping system. It is important to note that this study was conducted over a short period of three years. However, with the continuous improvement in soil health, it is possible to achieve significantly higher yields from MT in the long run.

#### 3.1.5 Three-year combined impact of nutrient management on crop yield

After conducting experiments over a span of three years, the analysis of the yield data demonstrated that the utilization of various nutrient management strategies had a positive impact on the productivity of all the crops within the cropping system, as indicated in Table 7. Specifically, when examining the performance of mustard, it was observed that NM_3_ exhibited a significantly higher average seed yield (1.66 t ha^−1^) compared to the other nutrient management packages, with statistical significance at p ≤ 0.001. NM_2_ followed closely behind with a seed yield of 1.47 t ha ^−1^ while NM_1_ and NM_4_ achieved yields of 1.45 t ha^−1^ and 0.51 t ha^−1^, respectively. The occurrence may have occurred due to the utilization of organic amendment, which effectively enhances the carbon content of the soil and nitrogen pools. This, in turn, leads to the conservation of soil moisture, resulting in an increased abundance of microbes and the uptake of more available nutrients by crops from solution pools. A study conducted by Nayak et al. [53] investigated the mustard-rice cropping system and found that the rabi seasons' mustard yielded the highest average production when implementing the Integrated Plant Nutrition System (IPNS) package.

**Table 7:** Average yield (pooled) (t ha^−1^) of cultivated crops in different nutrient management over three seasons (2018–2021).

Nutrient management	Mustard	Mungbean	T. aus	T. aman
NM_1_	1.45 b	1.00 b	3.32 b	3.90 b
NM_2_	1.47 b	1.05 b	3.64 a	3.97 b
NM_3_	1.66 a	1.22 a	3.60 a	4.43 a
NM_4_	0.51 c	0.59 c	2.56 c	2.63 c
CV (%)	8.94	11.2	9.24	10.30
LS	***	***	***	***

NM_1_ = 100 % STB dose (chemical fertilizer), NM_2_ = 125 % of STB dose (chemical fertilizer), NM_3_ = IPNS (80 % chemical fertilizer+ 20 % organic fertilizer), NM_4_ = Native fertility, *** = 0.1 % level of significant, LS = Level of significant, CV=Co-efficient of variation, T. aus = Transplanting aus, T. aman = Transplanting aman.

In the case of mungbean, the performance of NM_3_ was significantly superior (p ≤ 0.001) in terms of the three-year average seed yield (1.22 t ha^−1^) compared to NM_1_ (1.00 t ha^−1^), NM_2_ (1.05 t ha^−1^), and NM_4_ (0.59 t ha^−1^) (Table 7). This exceptional performance can be attributed to the creation of a soil environment that is enriched with microbial activity through the integrated use of organic and inorganic fertilizer. This enhanced microbial activity facilitates the preparation of nutrients for crops through processes such as aerobic ammonia oxidation and nitrification. Additionally, the presence of residual nutrients from previous crops, in conjunction with the applied nutrients, contributes to the promotion of crop performance. In a study conducted by Singh et al. [54], it was found that the IPNS approach, which combines chemical fertilizer with vermicompost, resulted in the highest average seed yield for mungbean compared to alternative chemical fertilizer dosages. For the T. aus, the grain yield was significantly influenced (p ≤ 0.001) by different strategies for nutrient management over the years. Among these strategies, the NM_2_ package demonstrated the highest average yield over three years (3.64 t ha^−1^), which was statistically similar to NM_3_ (3.60 t ha^−1^), but significantly higher than NM_1_ (3.32 t ha^−1^) and NM_4_ (2.56 t ha^−1^) (Table 7). Although NM_3_ ultimately resulted in the maximum yield due to a gradual improvement in soil fertility, NM_2_ initially achieved the highest yield, possibly due to favorable environmental conditions. Therefore, NM_2_ yielded slightly higher on average compared to NM_3_, with no statistical difference. According to Liu et al. [55], a study showed that while a combination of organic and inorganic nitrogen fertilizers resulted in the maximum rice grain yield in the final year, but the highest average rice grain yield was obtained from a combination of slow-release nitrogen and inorganic nitrogen fertilizers. After the completion of three consecutive years of experimentation, the grain yield of T. aman rice exhibited a significant impact when subjected to various nutrient packages (Table 7). In comparison to the treatments involving chemical fertilizers (NM_1_ = 3.90 t ha^−1^ and NM_2_ = 3.97 t ha^−1^) and native fertility (NM_4_ = 2.63 t ha^−1^), the organic-based NM_3_ yielded the highest average yield over the course of three years (4.43 t ha^−1^) with considerable significance (p ≤ 0.001). This can be attributed to the increase in carbon accumulation in the soil, which led to enhanced microbial biomass and reduced nutrient loss, ultimately resulting in higher crop yield. The absence of fertilizer application to compensate for NM_4_ resulted in nutrient deficiency in the crops, leading to a gradual decline in yield. In a study conducted by Gao et al. [56] on a rice-rice cropping system, long-term experimentation was carried out using solely organic, solely inorganic, and a combination of organic and inorganic fertilizers. The findings revealed that the combination of NPK fertilizer and cow manure exhibited the highest average year-round rice grain yield.

#### 3.1.6 Combination effect of year and tillage; year and nutrient on yield of cultivated crops

The interaction effect of tillage and year exhibited significant variation (p ≤ 0.05) solely for the mustard crop (Table 8). Amongst the tillage methods, MT yielded the highest mean yield for three consecutive years during 2020–21 (1.44 t ha^−1^), followed by CT during 2020–21 (1.35 t ha^−1^), and DT during 2020–21 (1.33 t ha^−1^). Conversely, MT during 2018–19 (1.13 t ha^−1^) resulted in the lowest mean yield, followed by CT during 2018–19 (1.14 t ha^−1^). This outcome could be attributed to the consistent application of minimum soil disturbance over three years, which contributed to the stability of soil aggregates, reduced soil moisture loss, promoted the growth of microbial colonies, and enhanced microbial activity. Additionally, increased soil moisture facilitated the availability of soil nutrients for crop growth and development. Interestingly, the aforementioned findings align with the research conducted by Chen et al. [50] in the context of the toria-maize cropping system.

**Table 8:** Combination effect of tillage and year on average (pooled) yield (t ha^−1^) of mustard.

Year × Tillage operations	Mustard
2018–19 × MT	1.13 c
2018–19 × CT	1.14 c
2018–19 × DT	1.24 bc
2019–20 × MT	1.30 ab
2019–20 × CT	1.27 bc
2019–20 × DT	1.26 bc
2020–21 × MT	1.44 a
2020–21 × CT	1.35 ab
2020–21 × DT	1.33 ab
CV (%)	8.95
LS	*

MT = Minimum Tillage, CT=Conventional Tillage, DT = Deep Tillage, * = 5 % level of significant, LS = Level of significant, CV=Co-efficient of variation.

The interaction between the year and nutrient management exhibited a significant effect in the cultivation of mustard (p ≤ 0.001), mungbean (p ≤ 0.001), and T. aus (p ≤ 0.01), but not in T. aman (Table 9). In all of these crops, the IPNS (NM_3_) package demonstrated the highest mean yield over a period of three years during 2020–21 (mustard = 1.83 t ha^−1^, mungbean = 1.40 t ha^−1^, T. aus = 3.96 t ha^−1^, and T. aman = 4.62 t ha^−1^), compared to other year and nutrient combinations. This can be attributed to the long-term application of organic fertilizer, which enhanced the physical, chemical, and biological properties of the soil. Conversely, the native fertility (NM_4_) resulted in the lowest average yield over a three-year period (mustard = 0.44 t ha^−1^, mungbean = 0.50 t ha^−1^, T. aus = 2.46 t ha^−1^, and T. aman = 2.51 t ha^−1^) during 2020–21). This can be attributed to the lack of fertilizer application, leading to a deficiency in the required nutrients and the dissipation of stored soil nutrients over time. Similar findings were reported by Das et al. [49] in the context of the toria-maize cropping system.

**Table 9:** Combination effect of year and nutrient management on average (pooled) yield (t ha^−1^) of cultivated crops.

Year × Nutrient management	Mustard	Mungbean	T. aus	T. aman
2018–19 × NM_1_	1.29 e	0.88 e	3.17 d	3.75
2018–19 × NM_2_	1.33 de	0.92 de	3.46 bcd	3.76
2018–19 × NM_3_	1.48 bcd	1.00 cde	3.20 cd	4.22
2018–19 × NM_4_	0.59 f	0.68 f	2.65 e	2.78
2019–20 × NM_1_	1.46 cde	1.01 cd	3.32 cd	3.89
2019–20 × NM_2_	1.48 bcd	1.06 cd	3.64 abcd	4.00
2019–20 × NM_3_	1.66 ab	1.24 ab	3.65 abc	4.46
2019–20 × NM_4_	0.51 f	0.59 fg	2.56 e	2.59
2020–21 × NM_1_	1.60 bc	1.13 bc	3.47 bcd	4.07
2020–21 × NM_2_	1.61 bc	1.18 bc	3.82 ab	4.15
2020–21 × NM_3_	1.83 a	1.40 a	3.96 a	4.62
2020–21 × NM_4_	0.44 f	0.50 g	2.46 e	2.51
CV (%)	8.95	11.24	9.24	10.30
LS	***	***	**	NS

NM_1_ = 100 % STB dose (chemical fertilizer), NM_2_ = 125 % of STB dose (chemical fertilizer), NM_3_ = IPNS (80 % chemical fertilizer+ 20 % organic fertilizer), NM_4_ = Native fertility, *** = 0.1 % level of significant, ** = 1 % level of significant, NS= Non significant, LS = Level of significant, CV=Co-efficient of variation, T. aus = Transplanting aus, T. aman = Transplanting aman.

#### 3.1.7 Combination effect of tillage and nutrient on yield of cultivated crops

The interaction between tillage and nutrient management exhibited positive variations for mustard (p ≤ 0.05), mungbean (p ≤ 0.001), T. aus (p ≤ 0.01), and T. aman (p ≤ 0.05) (Table 10). Among these crops, mustard demonstrated that MT resulted in the highest average yield over a period of three years when implemented with NM_3_ package (1.72 t ha^−1^). This yield was statistically equivalent to that of DT with the NM_3_ package (1.65 t ha^−1^) and CT with the NM_3_ package (1.60 t ha^−1^). However, it was significantly greater than the other combinations of tillage and nutrient management. On the other hand, CT with NM_4_ resulted in the lowest average yield over three years (0.46 t ha^−1^). It may have transpired as both diminished soil disturbance and the addition of organic matter created a mutually beneficial scenario for diverse microbial activity, the retention of soil moisture, and the lessening of soil compaction in the presence of carbon-enriched topsoil. This finding was corroborated by Malatas et al. [57] when they conducted their research in an alfalfa-maize cropping system. In the case of mungbean, conventional tillage yielded the highest average production (1.27 t ha^−1^) within the framework of the Integrated Plant Nutrition System (IPNS) package (NM_3_), which is statistically equivalent to both deep tillage with NM_3_ (1.20 t ha^−1^) and minimum tillage with NM_3_ (1.18 t ha^−1^), but significantly superior to other combinations of tillage and nutrients. Conversely, conventional tillage with NM_4_ resulted in the lowest average yield over a span of three years (0.56 t ha^−1^). Das et al. [49] also observed the same outcome in maize when conventional tillage was combined with maize stalk, ambrosia, and poultry manure under the maize-toria cropping system. In the case of T. aus, the highest average yield (3.81 t ha^−1^) was achieved through DT with NM_2_ (125 % of STB dose), which is statistically similar to CT under NM_2_ (3.76 t ha^−1^). This was followed by CT NM_3_, MTNM_3_, CTNM_1_, and MTNM_2_ combinations, but significantly higher than other tillage nutrient management combinations. However, the lowest average yield over three years (2.38 t ha^−1^) was obtained through DT under NM_4_. Michael et al. [58] found that excessive tillage with fertilizer and residue resulted in a higher yield compared to zero tillage with fertilizer and residue, when the same amount of fertilizer and residue was used in maize. For T. aman, CT with NM_3_ performed the best average yield (4.66 t ha^−1^), which is statistically equivalent to DT with NM_3_ (4.46 t ha^−1^) and MT with NM_3_ (4.18 t ha^−1^), but significantly higher than other tillage and nutrient combinations. On the other hand, the lowest average yield over three years (2.46 t ha^−1^) was achieved through DT under NM_4_. Das et al. [49] also stated that the highest average maize yield was obtained through conventional tillage with a combination of maize stalk, ambrosia, and poultry manure.

**Table 10:** Interaction effect of tillage and nutrient management on yield (pooled) (t ha^−1^) of different crops (2018–2021).

Tillage operations × Nutrient management	Mustard	Mungbean	T. aus	T.aman
MT × NM_1_	1.48 bcd	1.07 bc	3.26 cd	3.85 b
MT × NM_2_	1.40 d	0.98 cd	3.36 abc	3.91 b
MT × NM_3_	1.72 a	1.18 ab	3.56 abc	4.18 ab
MT × NM_4_	0.56 e	0.56 c	2.82 de	2.95 c
CT × NM_1_	1.41 d	1.08 bc	3.43 abc	3.98 b
CT × NM_2_	1.54 abcd	1.04 bc	3.76 a	4.10 b
CT × NM_3_	1.60 abc	1.27 a	3.75 ab	4.66 a
CT × NM_4_	0.46 e	0.60 c	2.48 e	2.47 c
DT × NM_1_	1.46 cd	0.86 d	3.27 bcd	3.88 b
DT × NM_2_	1.48 bcd	1.13 abc	3.81 a	3.91 b
DT × NM_3_	1.65 ab	1.20 ab	3.50 abc	4.46 ab
DT × NM_4_	0.52 e	0.61 e	2.38 e	2.46 c
CV (%)	8.95	11.24	9.24	10.30
LS	*	***	**	*

MT = Minimum tillage, CT=Conventional tillage, DT = Deep tillage, NM_1_ = 100 % STB dose (chemical fertilizer), NM_2_ = 125 % of STB dose (chemical fertilizer), NM_3_ = IPNS (80 % chemical fertilizer+ 20 % organic fertilizer), NM_4_ = Native fertility, *** = 0.1 % level of significant, ** = 1 % level of significant, * = 5 % level of significant LS = Level of significant, CV=Co-efficient of variation, T. aus = Transplanting aus, T. aman = Transplanting aman.

### 3.2 Three years impact of tillage and nutrient management on soil health and crop productivity

#### 3.2.1 Impact on crop productivity

##### 3.2.1.1 Impact of tillage on REY, SP and PE

Tillage had significant impact (p ≤ 0.05) on rice equivalent yield (REY), system productivity (SP) and production efficiency (PE) (Table 11). Although statistically identical to CT (REY = 13.99 t ha^−1^, SP = 9.77 t ha^−1^, and PE = 28.25 kg ha^−1^ day^−1^), the MT substantially produced the highest REY (14.32 t ha^−1^), SP (9.93 t ha^−1^), and PE (28.69 kg ha^−1^ day^−1^) over DT (REY = 13.46 t ha^−1^, SP = 9.37 t ha^−1^, and PE = 27.07 kg ha^−1^ day^−1^). Salahin et al. [24] asserted that strip tillage, as opposed to conventional tillage, exhibited superior performance in terms of rice equivalent yield. According to Kumar et al. [59], the rice-fallow-pulse/oilseed cropping system revealed that reduced tillage showcased the highest level of system productivity in comparison to conventional tillage.

**Table 11:** Mean rice equivalent yield, system productivity and production efficiency after three years of tillage and nutrient management.

Tillage operations	REY	SP	PE (kg ha^−1^ day^−1^)
	t ha^−1^		
MT	14.32 a	9.93 a	28.69 a
CT	13.99 ab	9.77 ab	28.25 ab
DT	13.46 b	9.37 b	27.07 b
CV (%)	5.06	5.25	5.25
LS	*	*	*
Nutrient management			
NM_1_	15.01 b	10.27 b	29.70 b
NM_2_	15.60 b	10.76 b	31.10 b
NM_3_	17.46 a	11.81 a	34.14 a
NM_4_	7.63 c	5.90 c	17.09 c
CV (%)	5.06	5.25	5.25
LS	***	***	***

REY= Rice equivalent yield, SP= System productivity, PE= Production efficiency, NM_1_ = 100 % STB dose (chemical fertilizer), NM_2_ = 125 % of STB dose (chemical fertilizer), NM_3_ = IPNS (80 % chemical fertilizer+ 20 % organic fertilizer), NM_4_ = Native fertility, MT = Minimum Tillage, CT=Conventional Tillage, DT = Deep Tillage, NS= Non significant, * = 5 % level of significant, *** = 0.1 % level of significant, LS = Level of significant, CV=Co-efficient of variation.

##### 3.2.1.2 Impact of nutrient management on REY, SP and PE

Rice equivalent yield (REY), system productivity (SP) and production efficiency (PE) were significantly varied (p ≤ 0.001) with nutrient managements (Table 11). IPNS (NM_3_) significantly performed the best result (REY = 17.46 t ha^−1^, SP = 11.81 t ha^−1^ and PE = 34.14 kg ha^−1^ day^−1^) in relation to others nutrient managements (NM_1_, NM_2_ and NM_4_) where the chemical fertilizer treatments (For NM_1_; REY = 15.01 t ha^−1^, SP = 10.27 t ha^−1^ and PE = 29.7 kg ha^−1^ day^−1^ and for NM_2_; REY = 15.6 t ha^−1^, SP = 10.76 t ha^−1^ and PE = 31.10 kg ha^−1^ day^−1^) resulted no discernible variation and native fertilizer (NM_4_) plot performed the lowest result (REY = 7.63 t ha^−1^, SP = 5.9 t ha^−1^ and PE = 17.09 kg ha^−1^ day^−1^). According to Salahin et al. [24], it was found that the combination of inorganic and organic compounds yielded better results in terms of rice equivalent production compared to various doses of chemical fertilizers. Rahman et al. [60] also stated that both organic and inorganic fertilizers enhance the productivity of the mustard-boro-T. aman rice and maize-jute-T. aman rice cropping system. Furthermore, Islam et al. [20] observed that a cropping system based on four crops exhibited higher production efficiency compared to a cropping system based on three crops.

#### 3.2.2 Impact on soil health

##### 3.2.2.1 Impact of tillage on FC, BD and PR

Field capacity (FC), bulk density (BD) and penetration resistance (PR) did not differ significantly with the tillage techniques (Table 12). The MT contained the highest FC (32.4 %) followed by CT (31.6 %) and DT (31.5 %). The reason behind this phenomenon may be that minimal soil disturbance contributes to the stability of soil aggregates. Consequently, these aggregates facilitate the deposition of carbon into the soil by slowing down carbon emission, while also enhancing water storage. In addition to this, the minimal manipulation of soil hinders soil evaporation. The outcome of these observations bears a resemblance to the findings of Acar et al. [61]. On the other hand, CT produced the highest BD (1.49 g cc-^1^) and PR (245 N cm^−2^) followed by MT (BD = 1.47 g cc-^1^ and PR = 241 N cm^−2^) and DT (BD = 1.46 g cc-^1^ and PR (234 N cm^−2^) due to soil consolidation in the subsoil for continuous excessive tillage though passing of heavy machine. On the other hand, continuous deep tillage made the soil slightly soft. According to Salahin et al. [62] and Alam et al. [63], there is no difference in bulk density between minimum, conventional, and deep tillage, and deep tillage exhibits the least amount of bulk density.

**Table 12:** Mean value of FC, BD and PR after three years of tillage and nutrient management.

Tillage operations	FC (%)	BD (g cc^−1^)	PR (N cm^−2^)
MT	32.4	1.47	241
CT	31.6	1.49	245
DT	31.5	1.46	234
CV (%)	3.12	1.43	9.37
LS	NS	NS	NS
Nutrient management			
NM_1_	31.8 b	1.49 a	245 a
NM_2_	32.0 b	1.48 a	241 a
NM_3_	33.4 a	1.42 b	203 b
NM_4_	30.2 c	1.51 a	270 a
CV (%)	3.12	1.43	9.38
LS	***	***	***

NM_1_ = 100 % STB dose (chemical fertilizer), NM_2_ = 125 % of STB dose (chemical fertilizer), NM_3_ = IPNS (80 % chemical fertilizer+ 20 % organic fertilizer), NM_4_ = Native fertility, MT = Minimum Tillage, CT=Conventional Tillage, DT = Deep Tillage, FC= Field capacity, BD= Bulk density, PR= Penetration resistance, NS= Non significant, *** = 0.1 % level of significant, LS = Level of significant, CV=Co-efficient of variation.

##### 3.2.2.2 Impact of nutrient management on FC, BD and PR

Field capacity (FC), bulk density (BD) and penetration resistance (PR) varied significantly due to different nutrient management practices (Table 12). Organic dominant nutrient package (NM_3_) significantly gave the maximum FC (33.4 %) together with the least BD (1.42 g cc^−1^) and PR (203 N cm^−2^) compare to sole chemical based nutrient management packages (For NM_1_; FC = 31.8 %, BD = 1.49 g cc^−1^ and PR = 245 N cm^−2^ and for NM_2_; FC = 32.0 %, BD = 1.48 g cc^−1^ and PR = 241 N cm^−2^). This might have occurred because made organic matter for organic fertilizer application, improved soil configuration resulting hold more soil water in the soil pores, reduced surface runoff. The NM_4_ plot had the worst results (FC = 30.2 %, BD = 1.51 g cc^−1^, and PR = 270 N cm^−2^). This could potentially be attributed to the absence of fertilizer supplementation, resulting in inadequate crop performance and an insufficient amount of biomass to sufficiently cover the soil. Ultimately, the intensifying heat from the scorching sunlight expedited soil compaction. In a study focused on rice-based cropping systems, Sandhu et al. [64] discovered that incorporating organic amendments such as cowdung and/or poultry manure, alongside inorganic fertilizer, enhanced field capacity and reduced bulk density. Similarly, Alam et al. [6] documented that the utilization of organic matter effectively decreased penetration resistance.

##### 3.2.2.3 Impact of tillage on pH, OM, TOC, MBC, total N and MBN

Tillage practices had no significant effect on pH, organic matter (OM), total organic carbon (TOC) and microbial biomass carbon (MBC) (Table 13). Comparatively more organic matter accumulated in MT soil than CT and DT soil as less soil disturbance reduced soil degradation resulting reduced carbon decomposition rate, making it superior in terms of pH (5.84), OM (1.38 %), TOC (17.7 t ha^−1^), and MBC (249 μg g^−1^) to other tillage regimes (For CT; pH = 5.80, OM = 1.34 %, TOC = 17.3 t ha^−1^, and MBC = 236 μg g^−1^ and for DT; pH = 5.81, OM = 1.32 %, TOC = 16.8 t ha^−1^, and MBC = 227 μg g^−1^). The current research is consistent with Salahin et al. [5] and Alam et al. [13]. Total nitrogen (TN) did not vary, while microbial biomass nitrogen (MBN) was significantly impacted by tillage methods (Table 13). MT, out of all the tillage techniques, produced the highest values for both TN (0.071 %) and MBN (19.1 μg g^−1^) while DT produced the lowest TN (0.062 %) and MBN (15.0 μg g^−1^). Minimum soil disturbance resulted in the deposition of a greater amount of organic carbon in the top layer of soil. This consequently led to an increase in the availability of nutrients, particularly nitrogen fertilizer, due to the enhanced microbial activity. The aforementioned discovery corroborates the findings of Chichongue et al. [12].

**Table 13:** Mean value of soil pH, OM, TOC, MBC, TN, MBN, P, K, S, Zn and B after three years of tillage and nutritional management.

Tillage operations	pH	OM	TN	TOC (t ha^−1^)	K (meq 100 g^-1^)	MBC	MBN	P	S	Zn	B
		%				μg g^−1^					
MT	5.84	1.38	0.071	17.7	0.13	249	19.1 a	16.2	15.8	1.42	0.22
CT	5.80	1.34	0.068	17.3	0.12	236	15.7 b	15.0	14.4	1.39	0.21
DT	5.81	1.32	0.062	16.8	0.12	227	15.0 b	14.8	14.2	1.38	0.20
CV (%)	3.75	7.48	15.27	7.06	11.8	10.9	15.7	10.3	14.7	14.4	11.4
LS	NS	NS	NS	NS	NS	NS	**	NS	NS	NS	NS
Nutrient management											
NM_1_	5.79 b	1.34 b	0.067 b	17.4 b	0.12 b	240 b	15.1 b	15.6 b	15.0 b	1.42 b	0.24 b
NM_2_	5.80 b	1.36 b	0.068 b	17.6 b	0.13 b	243 b	15.6 b	15.8 b	15.5 b	1.44 b	0.25 b
NM_3_	6.13a	1.73 a	0.091 a	21.3 a	0.17 a	288 a	28.0 a	21.2 a	18.9 a	1.69 a	0.28 a
NM_4_	5.52 c	0.96 c	0.041 c	12.7 c	0.07 c	179 c	7.8 c	8.7 c	9.7 c	1.03 c	0.08 c
CV (%)	3.75	7.48	15.27	7.06	11.8	10.9	15.71	10.3	14.7	14.3	11.4
LS	***	***	***	***	***	***	***	***	***	***	***

NM_1_ = 100 % STB dose (chemical fertilizer), NM_2_ = 125 % of STB dose (chemical fertilizer), NM_3_ = IPNS (80 % chemical fertilizer+ 20 % organic fertilizer), NM_4_ = Native fertility, MT = Minimum Tillage, CT=Conventional Tillage, DT = Deep Tillage, OM= Organic matter, TOC = Total organic carbon, MBC = Microbial biomass carbon, MBN = Microbial biomass nitrogen, TN = Total nitrogen, P= Phosphorus, K=Potassium, S=Sulphur, Zn = Zinc, B=Boron, ** = 1 % level of significant *** = 0.1 % level of significant, NS= Non significant, LS = Level of significant, CV=Co-efficient of variation.

##### 3.2.2.4 Impact of nutrient management on pH, OM, TOC, MBC, total N and MBN

According to Table 13, various nutrition managements led to significant variations in pH, organic matter (OM), total organic carbon (TOC), and microbial biomass carbon (MBC). When compared to other nutrient packages (NM_1_, NM_2_ and NM_4_) the organic-based nutrient management package (NM_3_) significantly produced the best results (pH = 6.13, OM = 1.73 %, TOC = 21.3 t ha^−1^ and MBC = 288 μg g^−1^) because adding organic manure aided in reserving more organic carbon into the soil, better soil microbe colonies were created, which sped up the soil reaction. There was no discernible difference between the two doses of chemical fertilizer (For NM_1_; pH = 5.79, OM = 1.34 %, TOC = 17.4 t ha^−1^ and MBC = 240 μg g^−1^ and for NM_2_; pH = 5.80, OM = 1.36 %, TOC = 17.6 t ha^−1^ and MBC = 243 μg g^−1^). Native fertile (NM_4_) plot gave the lowest result (pH = 5.52, OM = 0.96 % TOC = 12.7 t ha^−1^ and MBC = 179 μg g^−1^). When employing diverse organic amendments on rice and wheat, Hammad et al. [65] ascertained that the soil's pH experienced a substantial increase upon the combination of chemical fertilizer and cattle manure. According to Meena et al. [66], both organic and inorganic fertilizers made a notable contribution to the highest microbial biomass carbon (MBC). Bhardwaz et al. [18] unveiled that the implementation of an integrated plant nutrition system exerted a positive impact on the soil's organic matter. MBN along with TN was varied with different nutrient management (Table 13). NM_3_ significantly gave the highest MBN (28.0 μg g^−1^) and total N (0.091 %) than sole chemical fertilizers (For NM_1_; MBN = 15.1 μg g^−1^ and total N = 0.067 % and for NM_2_; MBN = 15.6 μg g^−1^ and total N = 0.068 %) and native fertility (NM_4_ = MBN = 7.8 μg g^−1^ and total N = 0.041 %). This might have happened because organic fertilizer increased the amount of organic carbon in the soil, which increased nutrient mineralization for microbial richness and activity. The results are consistent with those mentioned by Luo et al. [67].

##### 3.2.2.5 Impact of tillage on soil nutrient elements (P, K, S, Zn and B)

Tillage strategies have minimal impact on soil nutrients such P, K, S, Zn, and B. (Table 13). After completion of three cycles of cultivation, MT comparatively contained the more P (16.2 μg g^−1^), K (0.13 meq 100 g^−1^), S (15.8 μg g^−1^), Zn (1.42 μg g^−1^) and B (0.22 μg g^−1^) compare to other two tillages (For CT; P = 15.0 μg g^−1^, K = 0.12 meq 100 g^−1^, S = 14.4 μg g^−1^, Zn = 1.39 μg g^−1^ and B = 0.21 μg g^−1^ and for DT; P = 14.8 μg g^−1^, K = 0.12 meq 100 g^−1^, S = 14.2 μg g^−1^, Zn = 1.38 μg g^−1^ and B = 0.20 μg g^−1^) as less soil disturbance promotes better soil carbon stabilization, which in turn encourages a variety of soil microorganisms and aids in the mineralization of nutrients. On the other hand, excessive puddling may have increase surface runoff by blocking soil pores in the subsoil, which causes nutrients to drain from the soil. In addition, excessive tillage may retard mineralization because of low microbial activity. According to Salahin et al. [5], under the oilseed-pulse-rice cropping system, strip tillage maintained greater P, K, and B in post-harvest soil compared to conventional tillage. Reduced tillage enhanced the amount of P, S, Zn, and B in the soil, according to Chen et al. [50].

##### 3.2.2.6 Impact of nutrient management on soil nutrients (P, K, S, Zn and B)

Different fertilizer management techniques resulted in considerable variations in soil nutrients including P, K, S, Zn, and B (Table 13). For all nutrients, combination of organic and inorganic fertilizer (NM_3_) significantly performed the maximum P (21.2 μg g^−1^), K (0.17 meq 100 g^−1^), S (18.9 μg g^−1^), Zn (1.69 μg g^−1^) and B (0.28 μg g^−1^) compare to sole chemical fertilizer doses (For NM_1_; P = 15.6 μg g^−1^, K = 0.12 meq 100 g^−1^, S = 15.0 μg g^−1^, Zn = 1.42 μg g^−1^ and B = 0.24 μg g^−1^ and for NM_2_; P = 15.8 μg g^−1^, K = 0.13 meq 100 g^−1^, S = 15.5 μg g^−1^, Zn = 1.44 μg g^−1^ and B = 0.25 μg g^−1^) after three years. Due to the dynamism of the soil microorganisms, nutrients were more readily available in the organic dominating plot than in chemically treated plots. The NM_4_ (P = 8.7 μg g^−1^, K = 0.07 meq 100 g^−1^, S = 9.7 μg g^−1^, Zn = 1.03 μg g^−1^ and B = 0.08 μg g^−1^) had the worst results because crops ran out of nutrients while trying to meet their biological needs. The results are consistent with Sandhu et al. [63] on the rice-wheat cropping system.

##### 3.2.2.7 Combination effect of tillage practices and nutrient management on soil particle size distribution

**Fig. 2:**
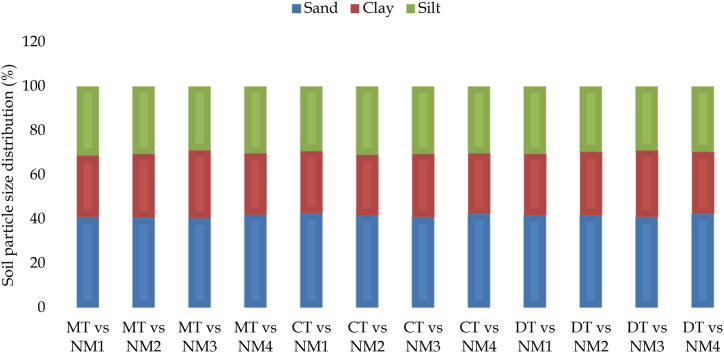
Combined effect of tillage and nutrient management on distribution soil particles. NM_1_ = 100 % STB dose (chemical fertilizer), NM_2_ = 125 % of STB dose (chemical fertilizer), NM_3_ = IPNS (80 % chemical fertilizer+ 20 % organic fertilizer), NM_4_ = Native fertility, MT = Minimum Tillage, CT=Conventional Tillage and DT = Deep Tillage.

No significant progress was made in altering the composition of the soil's different particle sizes when the practices of tillage and fertilizer management were combined (Fig. 2). Any attempt to manage the soil will face difficulties in modifying its texture, but a long-term strategy focusing on organic management has the potential to influence the quantity of soil particles due to the binding properties of organic matter. It was observed that tillage combined with IPNS (NM_3_) treatment resulted in the soil containing the highest proportion of clay particles and the lowest proportion of sand particles. Specifically, MT with NM_3_ treatment showed the highest clay particle content and the lowest sand particle content. This could be attributed to the continuous minimal manipulation of the soil, which contributed to the stability of soil aggregates and the preservation of carbon content. Additionally, the application of organic fertilizer increased carbon storage, leading to a strong bond between carbon and soil particles. In a study conducted by Salahin et al. [62], it was found that the texture of clay loam soil remained constant after soil management.

##### 3.2.2.8 Combination effect of tillage practices and nutrient management on soil moisture retention

**Fig. 3:**
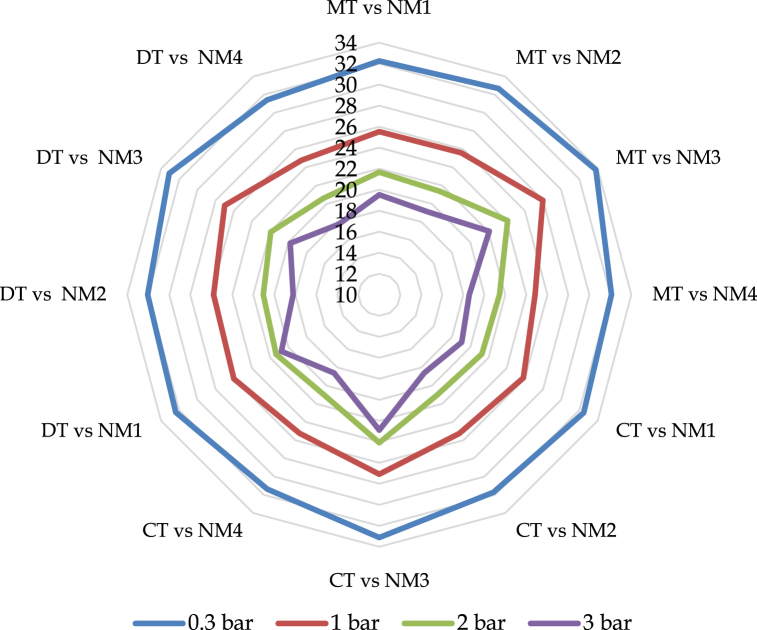
Combined effect of tillage and nutrient management impact on soil moisture retention at different bars (0.3, 1, 2 and 3). NM_1_ = 100 % STB dose (chemical fertilizer), NM_2_ = 125 % of STB dose (chemical fertilizer), NM_3_ = IPNS (80 % chemical fertilizer+ 20 % organic fertilizer), NM_4_ = Native fertility, MT = Minimum Tillage, CT=Conventional Tillage and DT = Deep Tillage.

Fig. 3 exemplified that there were no notable fluctuations in the amalgamation of treatments for moisture content at various bars (0.3, 1, 2, and 3). Tillage performed with a nutrient package based on organic substances (NM_3_) exhibited greater soil moisture at different bars, particularly MT with NM_3_, which retained the highest level of soil moisture. This occurrence can be attributed to the fact that less disturbed soil led to the stabilization of soil organic carbon and a reduction in soil evaporation. Additionally, the application of organic fertilizer aided in the accumulation of more soil organic carbon on the soil surface, thereby enhancing soil porosity. This mutually beneficial situation resulting from these two factors ultimately elevated the level of soil moisture. As stated by Islam et al. [68], there was no substantial disparity between conventional tillage and minimum tillage, despite the fact that minimum tillage retained significantly more soil moisture. Continuous utilization of cattle manure, pig manure, and wheat straw in conjunction with chemical fertilizer has a favorable effect on the enhancement of soil moisture retention, as reported by Zhou et al. [69].

## 4 Conclusion

In conclusion, the investigation conducted on tillage methodologies and integrated fertilizer approaches conducted in grey terrace soil highlighted the necessity of employing minimal tillage and integrated nutrient management to address the challenges associated with inadequate soil fertility and sustainable crop productivity. In the Mustard-Mungbean-T. aus-T. aman cropping system, findings revealed that minimal tillage surpassed deep tillage and conventional tillage in terms of crop yield, rice equivalent yield, system productivity, and production efficiency across all crops.

In terms of agricultural productivity, the Integrated Plant Nutrition System package (IPNS) also exhibited superior performance. The utilization of integrated nutrient management approaches and minimum tillage techniques yields a multitude of advantageous repercussions on the soil. These techniques enhance the field's capacity, augment the presence of organic matter within the soil, amplify the levels of microbial biomass carbon and nitrogen, as well as bolster the nutrient content within the soil. In pooled effect, minimum tillage showed the highest average yield solely for mustard, while conventional tillage resulted in the highest average yield for the remaining three crops. Over three years of study, minimum tillage has proven optimal for addressing soil concerns while maintaining yield. To accurately assess the effectiveness of minimum tillage, a long-term cropping system trial centered on this method is crucial for grasping the actual scenario. In terms of average yield across all crops, the Integrated Plant Nutrition System package (IPNS) outperformed other approaches. These findings signify the potential to heighten soil fertility and crop productivity, through the amalgamation of minimum tillage and integrated nutrient management. Consequently, these conclusions underscore the significance of implementing sustainable agricultural techniques to meet the escalating food demands of an expanding population, while simultaneously safeguarding the soil resources.

## Data availability statement

Data will be made available on request.

## CRediT authorship contribution statement

**Md. Jahangir Alam:** Writing - review & editing, Writing - original draft, Validation, Supervision, Project administration, Methodology, Investigation, Conceptualization. **Mahammad Shariful Islam:** Supervision, Investigation. **A.T.M. Anwarul Islam Mondol:** Supervision, Investigation. **Habib Mohammad Naser:** Supervision, Investigation. **Nazmus Salahin:** Supervision, Investigation. **Md. Khairul Alam:** Supervision, Investigation. **Md. Mazadul Islam:** Formal analysis. **Sanjida Akter:** Formal analysis. **Zakaria Alam:** Writing - review & editing, Writing - original draft, Visualization, Formal analysis, Data curation.

## Declaration of competing interest

The authors declare that they have no known competing financial interests or personal relationships that could have appeared to influence the work reported in this paper.
